# Proceedings of the American Society for Enhanced Recovery/Evidence Based Peri-Operative Medicine 2016 Annual Congress of Enhanced Recovery and Perioperative Medicine: Washington, D.C., USA, 20–22 April 2016

**DOI:** 10.1186/s13741-016-0045-0

**Published:** 2016-09-05

**Authors:** Charles R. Horres, Mohamed A. Adam, Zhifei Sun, Julie K. Thacker, Timothy J. Miller, Stuart A. Grant, Jeffrey Huang, Kirstie McPherson, Sanjiv Patel, Su Cheen Ng, Denise Veelo, Bart Geerts, Monty Mythen, Su Cheen Ng, Mark Foulger, Tim Collins, Kirstie McPherson, Michael Mythen, Mark Edwards, Denny Levett, Tristan Chapman, Imogen Fecher Jones, Julian Smith, John Knight, Michael Grocott, Mark Edwards, Thomas Sharp, Sandy Jack, Tom Armstrong, John Primrose, Michael Grocott, Denny Levett, Adam B. King, Kye Higdon, Melissa Bellomy, Sandy An, Paul St. Jacques, Jon Wanderer, Matthew McEvoy, Anne C. Fabrizio, Michael C. Grant, Deborah Hobson, Jonathan Efron, Susan Gearhart, Bashar Safar, Sandy Fang, Christopher Wu, Elizabeth Wick, Leanne Darwin, John Moore, Aparna Rege, Jayanth Reddy, William Irish, Ahmad Zaaroura, Elizabeth Flores Vera, Deepak Vikraman, Todd Brennan, Debra Sudan, Kadiyala Ravindra, Deborah Watson, Manasee V. Shah, Brett A. Maiese, Michael T. Eaddy, Orsolya Lunacsek, An Pham, George J. Wan, Kirstie McPherson, Thomas Keen, Monty Mythen, Alexander B Stone, Christopher L. Wu, Elizabeth C. Wick, Rachel A. Anolik, Adam Glener, Thomas J. Hopkins, Scott T. Hollenbeck, Julie K. Marosky Thacker, Tracey Hong, Andrea Bisaillon, Peter Black, Alan So, Kelly Mayson, Kirstie McPherson, Thomas Keen, Monty Mythen, Adam B. King, Rachel Forbes, Brad Koss, Tracy McGrane, Warren S. Sandberg, Jonathan Wanderer, Matthew McEvoy, Patrick Shanahan, John Rohan, Desirée Chappell, Carrie Chesher, Susan VanderBeek, Rebekah Kelly, Siamak Daneshmand, Soroush T. Bazargani, Hamed Ahmadi, Gus Miranda, Jie Cai, Anne K. Schuckman, Hooman Djaladat, Volz L., Milby J., Opeyemi Popoola, Tanisha Reid, Luciana Mullan, Mehrdad Rafizadeh, Richard Pitera

**Affiliations:** 1 Duke University School of Medicine, Durham, NC USA; 2 Anesthesilogist of Greater Orlando, Orlando, FL USA; 3 University College London, London, UK; 4 Academic Medical Center, Amsterdam, Netherlands; 5 University College London, London, UK; 6 Edwards Lifesciences, Irvine, CA USA; 7 William Harvey Hospital, Willesborough, Ashford, Kent UK; 8 University Hospital Southampton NHS Foundation Trust, Southhampton, UK; 9 University of Southampton, Southhampton, UK; 10 Royal College of Anaesthetists Health Services Research Centre, London, UK; 11 University Hospital Southampton NHS Foundation Trust, Southhampton, UK; 12 University of Southampton, Southhampton, UK; 13 Royal College of Anaesthetists Health Services Research Centre, London, UK; 14 Vanderbilt University Medical Center, Nashville, TN USA; 15 Johns Hopkins, Baltimore, MD USA; 16 North West Health Education England, Manchester, UK; 17 Central Manchester Foundation Trust, Manchester, UK; 18 Duke University Medical Center, Durham, NC USA; 19 CTI Clinical Trial and Consulting, Cincinnati, OH USA; 20 McGill University Health Centre, Montreal, Québec Canada; 21 Xcenda, Palm Harbor, FL USA; 22 Mallinckrodt Pharmaceuticals, Hazelwood, MO USA; 23 University College London Hospital, London, UK; 24 The Johns Hopkins Medical Institutions, Baltimore, MD USA; 25 Duke University School of Medicine, Durham, NC USA; 26 Vancouver General Hospital, Vancouver, British Columbia Canada; 27 University of British Columbia, Vancouver, British Columbia Canada; 28 University College London Hospital, London, UK; 29 Vanderbilt University Medical Center, Nashville, TN USA; 30 Anesthesiologists Consultants Enterprises, PLLC, Louisville, KY USA; 31 Norton Audubon Hospital, Louisville, KY USA; 32 Beaumont Health System, Troy, MI USA; 33 Keck Hospital of USC, Los Angeles, CA USA; 34 NYU Langone Medical Center, New York City, NY USA; 35 Oregon Health and Science University, Portland, OR USA; 36 University of Southern California, Los Angeles, CA USA; 37 Thompson J Dunes Surgical Hospital, Dakota Dunes, SD USA; 38 Saint Barnabas Medical Center, Livingston, NJ USA

## Abstract

A1 Effects of enhanced recovery pathways on renal function

Charles R. Horres, Mohamed A. Adam, Zhifei Sun, Julie K. Thacker, Timothy J. Miller, Stuart A. Grant

A2 Economic outcomes of enhanced recovery after surgery (ERAS)

Jeffrey Huang

A3 What does eating, drinking and mobilizing after enhanced recovery surgery really mean?

Kirstie McPherson, Sanjiv Patel, Su Cheen Ng, Denise Veelo, Bart Geerts, Monty Mythen

A4 Intra-operative fluid monitoring practices

Su Cheen Ng, Mark Foulger, Tim Collins, Kirstie McPherson, Michael Mythen

A5 Development of an integrated perioperative medicine care pathway

Mark Edwards, Denny Levett, Tristan Chapman, Imogen Fecher – Jones, Julian Smith, John Knight, Michael Grocott

A6 Cardiopulmonary exercise testing for collaborative decision making prior to major hepatobiliary surgery

Mark Edwards, Thomas Sharp, Sandy Jack, Tom Armstrong, John Primrose, Michael Grocott, Denny Levett

A7 Effect of an enhanced recovery program on length of stay for microvascular breast reconstruction patients

Adam B. King, Kye Higdon, Melissa Bellomy, Sandy An, Paul St. Jacques, Jon Wanderer, Matthew McEvoy

A8 Addressing readmissions associated with an enhanced recovery pathway for colorectal surgery

Anne C. Fabrizio, Michael C. Grant, Deborah Hobson, Jonathan Efron, Susan Gearhart, Bashar Safar, Sandy Fang, Christopher Wu, Elizabeth Wick

A9 The Manchester surgical outcomes project: prevalence of pre operative anaemia and peri operative red cell transfusion rates

Leanne Darwin, John Moore

A10 Preliminary results from a pilot study utilizing ears protocol in living donor nephrectomy

Aparna Rege, Jayanth Reddy, William Irish, Ahmad Zaaroura, Elizabeth Flores Vera, Deepak Vikraman, Todd Brennan, Debra Sudan, Kadiyala Ravindra

A11 Enhanced recovery after surgery: the role of the pathway coordinator

Deborah Watson

A12 Hospitalization costs for patients undergoing orthopedic surgery treated with intravenous acetaminophen (IV-APAP) + IV opioids or IV opioids alone for postoperative pain

Manasee V. Shah, Brett A. Maiese, Michael T. Eaddy, Orsolya Lunacsek, An Pham, George J. Wan

A13 Development of an app for quality improvement in enhanced recovery

Kirstie McPherson, Thomas Keen, Monty Mythen

A14 A clinical rotation in enhanced recovery pathways and evidence based perioperative medicine for medical students

Alexander B Stone, Christopher L. Wu, Elizabeth C. Wick

A15 Enhanced recovery after surgery (ERAS) implementation in abdominal based free flap breast reconstruction

Rachel A. Anolik, Adam Glener, Thomas J. Hopkins, Scott T. Hollenbeck, Julie K. Marosky Thacker

A16 How the implementation of an enhanced recovery after surgery (ERAS) protocol can improve outcomes for patients undergoing cystectomy

Tracey Hong, Andrea Bisaillon, Peter Black, Alan So, Associate Professor, Kelly Mayson

A17 Use of an app to improve patient engagement with enhanced recovery pathways

Kirstie McPherson, Thomas Keen, Monty Mythen

A18 Effect of an enhanced recovery after surgery pathway for living donor nephrectomy patients

Adam B. King, Rachel Forbes, Brad Koss, Tracy McGrane, Warren S. Sandberg, Jonathan Wanderer, Matthew McEvoy

A19 Introduction and implementation of an enhanced recovery program to a general surgery practice in a community hospital

Patrick Shanahan, John Rohan, Desirée Chappell, Carrie Chesher

A20 “Get fit” for surgery: benefits of a prehabilitation clinic for an enhanced recovery program for colorectal surgical patients

Susan VanderBeek, Rebekah Kelly

A21 Evaluation of gastrointestinal complications following radical cystectomy using enhanced recovery protocol

Siamak Daneshmand, Soroush T. Bazargani, Hamed Ahmadi, Gus Miranda, Jie Cai, Anne K. Schuckman, Hooman Djaladat

A22 Impact of a novel diabetic management protocol for carbohydrate loaded patients within an orthopedic ERAS protocol

Volz L, Milby J

A23 Institution of a patient blood management program to decrease blood transfusions in elective knee and hip arthroplasty

Opeyemi Popoola, Tanisha Reid, Luciana Mullan, Mehrdad Rafizadeh, Richard Pitera

## A1 Effects of enhanced recovery pathways on renal function

### **Authors:**Charles R. Horres, Mohamed A. Adam, Zhifei Sun, Julie K. Thacker, Timothy J. Miller, Stuart A. Grant

#### Duke University School of Medicine, Durham, NC, USA

##### **Correspondence:**Charles R. Horres – Duke University School of Medicine, Durham, NC, USA

**Background**

Aggressive intravenous fluid replacement regimens are traditionally employed with the intention of protecting patients from perioperative decreases in renal blood flow. In contrast to these regimens, Enhanced Recovery Pathways (ERPs) often employ intraoperative goal-directed fluid therapy and postoperative fluid restriction with permissive oliguria. While ERPs have been proven to reduce physiologic stress and improve outcomes in general, their impact on postoperative renal function remains unknown.

**Methods**

Patients undergoing major colorectal surgery within an ERP (2/2010 to 3/2013) were compared with a matched-control group undergoing surgery without an ERP (10/2004-10/2007) at a single institution.

Multivariable regression models were employed to examine the effect of ERPs on the change in postoperative creatinine and incidence of acute kidney injury (based on the RIFLE criteria).

**Results**

A total of 1054 patients were included: 590 patients in the ERP group, and 464 patients in the control group. Patient age, gender and race were similar between groups. The ERP group more often had significant comorbidities (62 % ASA ≥3 vs. 40 % ASA ≥3, p < 0.001), non-benign indications for surgery (81 % vs. 74 %, p = 0.045), and more extensive surgery (48 % vs. 12 % proctectomy, p < 0.001) compared to control. Unadjusted median increase in postoperative creatinine was slightly higher in ERP vs. control (0.1 vs. 0 mg/dL, respectively). After multivariable regression adjustment, postoperative change in creatinine was similar in ERP vs. control (p = 0.25). Compared to control, ERP associated with similar rates of postoperative acute kidney insufficiency (3.7 % ERP vs. 3.7 %) and acute Kidney failure (0.8 % vs. 0.9 %).

**Conclusions**

Implementation of an ERP in colorectal surgery is not associated with a clinically significant increase in the level of perioperative creatinine change or an increased incidence of postoperative acute kidney injury. Further studies should be conducted to address the risks and benefits of ERP in other surgical populations.

## A2 Economic outcomes of enhanced recovery after surgery (ERAS)

### **Author:**Jeffrey Huang

#### Anesthesilogist of Greater Orlando, Orlando, FL, USA

**Introduction**

Enhanced recovery after surgery (ERAS) is standardized, coordinated, interdisciplinary perioperative care plans that incorporate evidence-based interventions to minimize surgical stress, improve physiologic and functional recovery, reduce complications, and thereby facilitate earlier discharge from the hospital. The benefits of ERAS have been also demonstrated in patients undergoing urological, gynecological, upper gastrointestinal, hepatobiliary, cardiac, and vascular surgery. ERAS programs can improve clinical outcomes, also are associated with a reduction in costs as a result of the reduction in LOS and morbidity. The cost effectiveness and economic benefits of implementation of ERAS program had been examined.

**Methods**

Literatures review was performed. The studies with cost saving data were included in the review. The results were shown in the table.

**Table:** 

Type of surgery	Cost saving	References
Colorectal surgery	mean savings of 1651€ ($2245 USD) per colorectal surgical patient	Lee L Ann Surg 2014; 259: 670–6
Bariatric surgery	The mean cost per patient was significantly lower in the ERAS group than in the historical group (14 836 NZ$ vs 27 700 NZ$).	Lemanu DP Br J Surg 2013; 100: 482–9.
Gastric surgery	The hospital costs were significantly less in the ERAS group than in the conventional group (WMD −505.87 dollars, 95 % CI, −649.91 to −361.84 dollars).	Yu Z Langenbecks Arch Surg 2014; 399: 85–92.
Gynecology	a 30-day cost savings of more than $7,600 USD per patient (18.8 % reduction).	Kalogera E Obstet Gynecol 2013; 122(2 Pt 1): 319–28
Esophageal surgery	The pathway-dependent cost saving per patient was €1055 and the overall cost saving per patient was €2013.	Lee L BJS 2013; 100(10):: 1326–34
Cardiac surgery	ERAS group was associated with a reduction in total hospital cost compared with those for the control group (€ 4,625 and € 5,441, respectively).	van Mastrigt GA Crit Care Med. 2006;34(1):65–75.

**Conclusion**

Economic data from multiple studies supported that ERAS can improve healthcare quality with lower cost.

## A3 What does eating, drinking and mobilizing after enhanced recovery surgery really mean?

### **Authors:**Kirstie McPherson^1^, Sanjiv Patel^1^, Su Cheen Ng^1^, Denise Veelo^2^, Bart Geerts^2^, Monty Mythen^1^

#### ^1^University College London, London, UK; ^2^Academic Medical Center, Amsterdam, Netherlands

##### **Correspondence:**Kirstie McPherson – University College London, London, UK

**Background/Introduction**

Enhanced Recovery Pathways (ERP’s) focus on the delivery of bundles of evidence-based care practices that expedite a patient’s recovery and return to normal function. Central to recovery is establishing early mobilization and enteral nutrition.

It is well recognized that there are barriers to successful implementation of ERP’s [1]. One of these includes successful dissemination of a pathway and the goals of treatment to a multidisciplinary team. We were interested to examine the opinions of a group of experts, when defining those basic principles of eating, drinking and mobilizing after surgery.

**Methods**

Individuals invited to an enhanced surgical recovery working group meeting in Europe (n = 13) were contacted by email in December 2015 to anonymously complete an online questionnaire that probed their literal understanding of the definitions of *eating*, *drinking* and *mobilizing* after surgery.

All members of the working group had agreed to participate at the meeting, with the intent that it was important to identify variation, and ultimately establish a consensus, and in what these definitions meant in clinical practice to different individuals.

**Results**

All individuals contacted (n = 13) completed the questionnaire. There was variation in defining each domain, (eating, drinking and mobilizing). For drinking, the greatest number to agree (53.8 %, n = 7/13) described this feature as “*return to baseline fluid intake”*. 15.4 % (n = 2/13) qualified this definition as *“sips of clear fluid”.* For eating, again there was wide variation in defining the term. *“Light solid intake (such as sandwich/fruit/yoghurt)”* was the most cited response, by (53.8 %, n = 7/13) respondents. The greatest variation in responses was seen in defining mobilization after surgery. No consensus was reached, with equal numbers (n = 2) defining this postoperative goal as either *“sitting out of bed”*, *“minimal steps”*, *“independent walking without assistance”* or *“return to baseline function”*.

**Conclusion**

This small “snapshot” of experts’ views demonstrates an unmet need to more clearly define the goals for patients after surgery.

Interestingly, there is significant variation amongst champions of enhanced recovery in defining those important terms of eating, drinking and mobilizing after surgery. From this, we might infer that the delivery of clear guidance to adopters of enhanced recovery practices may be compromised, with the effect of diluting the impact of patient’s realizing these important goals.

In our institutions, we recommend explicitly defining these goals for patients *and* healthcare professionals in both patient information and published pathways. By effectively disseminating clear information, we anticipate less ambiguity in the interpretation of ERP’s, leading to better understanding of the provenance of the pathway by all. Moreover, we feel these important and agnostic indicators of recovery should be at the very heart of the focused drive of ERP’s.

**References**

1. Pearsall EA, Meghji Z, Pitzul KB, et al. A qualitative study to understand the barriers and enablers in implementing an enhanced recovery after surgery program. *Ann Surg* 2015; 261(1):92–6

## A4 Intra-operative fluid monitoring practices

### **Authors:** Su Cheen Ng^1^, Mark Foulger^2^, Tim Collins^3^, Kirstie McPherson^1^, Michael Mythen^1^

#### ^1^University College London, London, UK; ^2^Edwards Lifesciences, Irvine, CA, USA; ^3^William Harvey Hospital, Willesborough, Ashford, Kent, UK

**Background/Introduction**

In the past, administration of intraoperative fluid therapy was guided by changes in blood pressure, heart rate, arterial waveform and central venous pressure. However these methods of measurements are neither sensitive nor specific. Now newer technologies are available to optimize haemodynamic status such as the esophageal Doppler, pulse pressure waveform analysis and changes in bioimpedance.

In the United Kingdom, many guidelines (GIFTASUP [1], NICE [2]) recommend the use of intraoperative fluid monitoring for surgical patients to achieve optimized perioperative fluid therapy. It forms an important element of goal directed therapy to tailor an individual’s fluid requirements to achieve central normovolaemia.

**Methods**

Two educational workshops were carried out for cardiac output (CO) monitoring during a perioperative medicine meeting. Participants were then asked 4 questions on their IOFM practices. A set of answers was listed and an audience participation system using keypad was used to sample the participants.

**Results**

The majority of participants (55 %, n = 40) would utilize CO monitoring during major risk cases or major surgery cases to guide their intra operative practice. Fifty five percent (n = 21) also stated they would attempt to incorporate IOFM into their routine practice.

**Fig. 1 (abstract A4).**
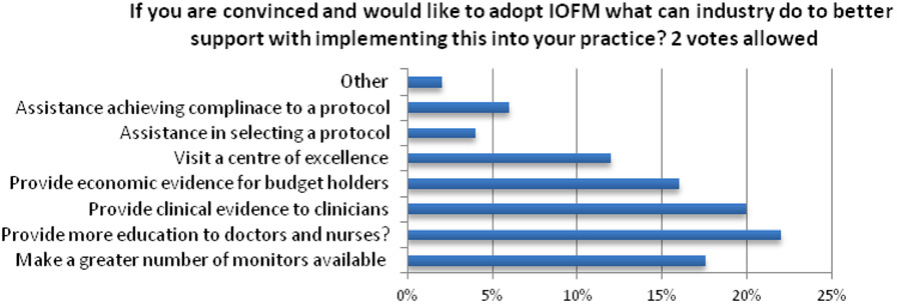
ᅟ

Among the participants (n = 26) who were not sure or unlikely to implement IOFM, the main reason was due to participants not being convinced with evidence for carrying out intraoperative monitoring (38 %) followed by the lack of agreement among peers (20 %). Only 12 % blamed lack of funding from their hospital or lack of equipment to carry out the practice. 22 % would adopt the practice if more education were provided (Fig. 1).

**Conclusion**

We have shown from the small cohort sampled that there are only a small proportion of anaesthetists that would not utilize CO optimization for moderate to high-risk surgical cases. Most clinicians therefore see the advantages of employing such practice. Education seems to be an important driving factor for the uptake of IOFM among the sampled cohort.

IOFM (and protocols) can lead to decreased variability of intraoperative fluid practices. The ultimate aim is to avoid excessive fluid restriction or overload in surgical patients, which has been associated with poorer outcomes [3].

**References**

1. Powell-Tuck J, Gosling P, Lobo DN, Allison SP, Carlson GL, Gore M, et al. British Consensus Guidelines on Intravenous Fluid Therapy for Adult Surgical Patients - GIFTASUP. 2008

2. National Institute for Health and Clinical Excellence. NICE medical technology guidance 3. (2011) Cardio-Q-ODM Oesophageal Doppler monitor

3. Perioperative Fluid Utilization Variability and Association With Outcomes: Considerations for Enhanced Recovery Efforts in Sample US Surgical Populations. Annals of Surgery, 263(3), pp 502–510

## A5 Development of an integrated perioperative medicine care pathway

### **Authors:**Mark Edwards^1,2,3^, Denny Levett ^1,2,3^, Tristan Chapman^1^, Imogen Fecher – Jones^1^, Julian Smith^1^, John Knight^1^, Michael Grocott^1,2,3^

#### **Affiliations:**^1^University Hospital Southampton NHS Foundation Trust, Southhampton, UK; ^2^University of Southampton, Southhampton, UK; ^3^Royal College of Anaesthetists Health Services Research Centre, London, UK

##### **Correspondence:**Mark Edwards – University Hospital Southampton NHS Foundation Trust, Southhampton, UK

**Background/Introduction**

Perioperative medicine is the patient-centered, multidisciplinary and integrated medical care of patients from the moment of contemplation of surgery until full recovery. It builds on the Enhanced Recovery approach to capitalize on five key opportunities: collaborative decision-making, preoperative lifestyle modification, standardization of perioperative care, achieving full postoperative recovery and using data to drive quality improvement [1]. We hypothesize that redesigning the perioperative pathway will add value through improved quality *and* reduced resource utilization.

**Methods**

Our multidisciplinary team is developing an integrated Perioperative Medicine care pathway at a large tertiary referral University hospital. Current pathways were mapped, analyzed and redesigned with particular focus on specific factors including defining the pathway boundaries, engaging patients and time constraints.

**Results**

**Fig. 2 (abstract A5).**
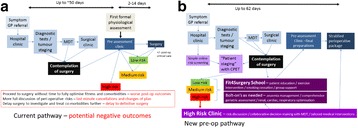
Proposed preoperative pathway redesign at University Hospital Southampton. MDT, multidisciplinary team

Current preoperative pathways were mapped and analyzed (see: Fig 2a). Pathway redesign addressed a number of specific aims (see: Fig. 2b): identification of the “moment of contemplation of surgery”, early targeted preoperative information gathering through a patient-driven online system; routine physiological assessment to stratify risk early in the preoperative pathway (“patient staging”) by cardiopulmonary exercise testing; a dedicated clinic for patients at high perioperative morbidity/mortality risk, collaborative decision-making and early medical optimization; Fit4Surgery School for all patients undergoing major surgery, targeting patient education, expectation management and lifestyle optimization; standardized perioperative management based on risk strata; postoperative care team clinical ward reviews for “at-risk” patients; postoperative electronic data capture to monitor Enhanced Recovery targets, morbidity, and patient-reported outcomes.

**Conclusion**

Perioperative medicine offers a unique opportunity to add value through improved outcomes and reduced resource utilization in patients undergoing major surgery. Extensive pathway redesign may be needed to ensure an integrated approach, maximizing the opportunities for improvements in preoperative optimization and postoperative care. Moving the evaluation of risk to a position earlier in the pre-operative pathway offers opportunities for risk mitigation, collaborative decision-making and optimization of patient health before surgery.

**References**

1. Grocott MPW, Mythen MG. Perioperative Medicine: The Value Proposition for Anesthesia?: A UK Perspective on Delivering Value from Anesthesiology. Anesthesiol Clin. 2015 Dec;33(4):617–28.

## A6 Cardiopulmonary exercise testing for collaborative decision making prior to major hepatobiliary surgery

### **Authors:**Mark Edwards^1,2,3^, Thomas Sharp^2^, Sandy Jack^1,2^, Tom Armstrong^1^, John Primrose^1,2^, Michael Grocott^1,2,3^, Denny Levett^1,2^

#### **Affiliations:**^1^University Hospital Southampton NHS Foundation Trust, Southhampton, UK; ^2^University of Southampton, Southhampton, UK; ^3^Royal College of Anaesthetists Health Services Research Centre, London, UK

##### **Correspondence:**Mark Edwards –^1^University Hospital Southampton NHS Foundation Trust, Southhampton, UK

**Background/Introduction**

Cardiopulmonary exercise testing (CPET) is increasingly used for preoperative risk assessment. Evidence to date suggests utility for predicting risk of postoperative morbidity and mortality across a number of surgical specialties [1]. It is commonly used to triage patients to postoperative critical care [2] and to inform preoperative risk discussions. We report its use for preoperative collaborative decision making in a large University hepatopancreatobiliary (HPB) surgical unit in which postoperative critical care admission is routine.

**Methods**

Patients undergoing assessment for liver resection and pancreaticoduodenectomy in 2014 and 2015 underwent symptom limited incremental exercise testing at the surgeons’ discretion. Data collected included anaerobic threshold (AT), peak oxygen consumption (peakVO_2_) and ventilatory equivalents for carbon dioxide at AT (VE/VCO_2_), clinical plan made on the basis of CPET, intensive care and hospital length of stay (LOS) in operated patients. Based on prior literature, physiological risk was reported to the clinical team as “low risk” (AT > 10mlO_2_.min^-1^.kg^-1^), “high risk” (AT 8-10mlO_2_.min^-1^.kg^-1^) or “very high risk” (AT <8mlO_2_.min^-1^.kg^-1^).

**Results**

146 patients underwent CPET. Median (IQR) age was 69 (62–74), with mean (SD) AT 9.6 (2.6) mlO_2_.min^-1^.kg^-1^. This is lower than previously published series of HPB patients [3] and may reflect selective referral of patients where the surgeon has concern about baseline physiological status. 31 patients did not ultimately have surgery. Of these 13 (8.9 %) had disease that was assessed as non-resectable whereas 18 (12.3 %) had very high physiological risk (mean AT 6.5 mlO_2_.min^-1^.kg^-1^; p < 0.0001 compared with operated group). Pursuing non-surgical treatment in these patients involved collaborative decision making between patient, surgeon, anesthesiologist and oncologist. Each potential treatment was explored in terms of benefits and risks, including the individualized risk level of postoperative morbidity / mortality suggested by CPET results. Alternative treatments included transarterial chemoembolization, chemotherapy, interval disease surveillance and palliative care.

Furthermore, in nine “high-/very high- risk” cases undergoing surgery (8 % of operated group), perioperative care was significantly modified based on CPET findings. This included four cases of optimization of cardiac medication for exercise-induced ischemia / arrhythmia and two respiratory interventions. This preoperative optimization group proceeded to surgery in a timely fashion (median time from test to surgery 9 days, range 1–20) and had postoperative outcomes in line with the lower risk CPET group: critical care LOS 1 day (range 1–6 days), hospital LOS 8 days (range 2–9 days).

**Conclusion**

Even in centers and surgical specialties where postoperative critical care admission is routine, preoperative CPET in a higher risk subset of the overall patient group has utility in guiding shared decision making. This includes consideration of non-surgical options in patients at very high risk of postoperative morbidity and mortality, and timely optimization of cardio-respiratory limitations revealed during CPET.

**References**

1. Hennis PJ, Meale PM, Grocott MPW. Cardiopulmonary exercise testing for the evaluation of perioperative risk in non-cardiopulmonary surgery. Postgrad Med J. 2011 Aug;87(1030):550–7.

2. Swart M, Carlisle JB. Case-controlled study of critical care or surgical ward care after elective open colorectal surgery. Br J Surg. 2012 Feb;99(2):295–9.

3. Snowden CP, Prentis J, Jacques B, Anderson H, Manas D, Jones D, et al. Cardiorespiratory Fitness Predicts Mortality and Hospital Length of Stay After Major Elective Surgery in Older People. Annals of Surgery. 2013 Jun;257(6):999–1004.

## A7 Effect of an enhanced recovery program on length of stay for microvascular breast reconstruction patients

### **Authors:**Adam B. King, Kye Higdon, Melissa Bellomy, Sandy An, Paul St. Jacques, Jon Wanderer, Matthew McEvoy

#### Vanderbilt University Medical Center, Nashville, TN, USA

##### **Correspondence:**Adam B. King – Vanderbilt University Medical Center, Nashville, TN, USA

**Introduction**

Perioperative care in the United States is often costly and fragmented, particularly for complex episodes of care. Our group has recently demonstrated how care redesign built upon enhanced recovery after surgery (ERAS) principles can decrease length of stay, postoperative complications, and cost of care for colorectal surgery patients.[1] However, there is little data surrounding such efforts for microvascular breast reconstruction patients.[2]

**Methods**

Following the same principles from earlier care redesign efforts [1], we implemented an ERAS pathway for all microvascular breast reconstruction patients in August 2015. After IRB approval, records were obtained for all elective microvascular breast reconstruction procedures performed for Phase 0 (2/1/2012-8/16/2015) and Phase 1 (8/17/2015 - 1/31/2016). Patient age and BMI were obtained along with intraoperative morphine equivalents. Case mix index and length of stay were abstracted from hospital billing records.

**Results**

154 charts were reviewed; 125 in Phase 1 and 29 in Phase 2. There were no differences in baseline characteristics between the two groups. Median length of stay was reduced in the ERAS group (4.36 vs 3.37, p = 0.002). Intraoperative morphine equivalents were also reduced in the ERAS group (44.71 vs 11.90, p < 0.001). Readmission rates were unchanged between the groups.

**Conclusion**

The ongoing ERAS pathway development by our Perioperative Consult Service for patients undergoing microvascular breast reconstruction significantly shortened median length of stay and decreased intraoperative opiate use while not affecting readmission rates.

**References**

1. McEvoy MD, Wanderer JP, King AB, Geiger TM, Tiwari V, Terekhov M, Ehrenfeld JM, Furman WR, Lee LA, Sandberg WS. A perioperative consult service results in reduction in cost and length of stay for colorectal surgical patients: evidence from a healthcare redesign project. *Perioperative medicine (London, England).* 2016;5:3.

2. Batdorf NJ, Lemaine V, Lovely JK, Ballman KV, Goede WJ, Martinez-Jorge J, Booth-Kowalczyk AL, Grubbs PL, Bungum LD, Saint-Cyr M. Enhanced recovery after surgery in microvascular breast reconstruction. *J Plast Reconstr Aesthet Surg.* 2015;68(3):395–402.

## A8 Addressing readmissions associated with an enhanced recovery pathway for colorectal surgery

### **Authors:**Anne C. Fabrizio, Michael C. Grant, Deborah Hobson, Jonathan Efron, Susan Gearhart, Bashar Safar, Sandy Fang, Christopher Wu, Elizabeth Wick

#### Johns Hopkins, Baltimore, MD, USA

##### **Correspondence:**Anne C. Fabrizio – Johns Hopkins, Baltimore, MD, USA

**Introduction**

Enhanced Recovery Pathways (ERPs) have gained favor in the United States as effective approaches to improve the quality and value of perioperative care. Most ERPs focus on pre-operative preparation, analgesia, fluid management and early mobility with a focus on improving performance on in-hospital metrics (length of stay and cost). Few ERPs include processes related to the hospital to home transfer and little has been reported regarding the rate and characteristic of patient readmission. We designed a study to determine the rate and reasons for readmissions in ERP vs. non-ERP patients and to identify areas to optimize ERP to prevent readmissions.

**Methods**

Patients enrolled in an ERP for colorectal surgery between February and December 2014 (ERP) were compared to a similar cohort of patients who received surgery prior to protocol implementation (preERP). Outcomes of interest included 30-day readmission rates, composite LOS, and readmission diagnosis.

**Results**

A total of 346 preERP and 330 ERP patients were included in the analysis. ERP was associated with a significant reduction in index hospitalization LOS (5.3 vs. 7.0 days; p < 0.001) and incidence of postoperative surgical site infection (SSI; 7.3 vs. 16.6 %; p = 0.013) compared to preERP. Rate of readmission within 30 days (17.6 vs. 19.4 %; p = 0.55) as well as mean time to readmission (9.0 vs. 8.7 days; p = 0.83) was similar between groups. As a result of similar readmission hospitalization LOS (5.7 vs. 5.2 days; p = 0.64), the composite hospital LOS was also similar between groups (12.0 vs. 13.5 days; p = 0.298). The table denotes the readmission diagnoses for each group, which a significant reduction in readmissions for SSI in the ERP group compared to preERP counterparts.

**Conclusion**

Although ERP did not lead to a reduction in hospital readmissions, patients received significant benefit through a reduction in index hospitalization length of stay and rates of postoperative SSI. To impact readmissions, teams should consider including care transition process measures into ERP. Common care transition process measures aimed at reducing readmission and improving patient outcomes such as the use of transition guides for high-risk patients, remote vital sign and symptom monitoring, early clinical follow up and post-discharge pharmacist follow up have not traditionally been part of ERP protocols. Incorporation of such measures into ERP has the potential to reduce rates of post-operative complication and readmissions particularly for high-risk patient populations.

**Table:** 

**Readmission Diagnosis**	**PreERP (n = 67)**	**ERP (n = 58)**	**P-value**
SBO/ileus	13 (19.1 %)	18 (31 %)	0.133
High output stoma	6 (9.0 %)	4 (6.9 %)	0.672
All SSI	34 (50.7 %)	17 (31 %)	*0.015*
Superficial/Deep SSI	16 (23.9 %)	6 (10.3 %)	*0.048*
Organ Space SSI	18 (26.9 %)	11 (19 %)	0.297
Thromboembolic event	0 (0 %)	3 (5.2 %)	0.060
Bleeding	0 (0 %)	2 (3.4 %)	0.125
Other	14 (20.9 %)	14 (24.1 %)	0.665

## A9 The Manchester surgical outcomes project: prevalence of pre operative anaemia and peri operative red cell transfusion rates

### **Authors:**Leanne Darwin^1^, John Moore^2^

#### ^1^North West Health Education England, Manchester, UK; ^2^Central Manchester Foundation Trust, Manchester, UK

##### **Correspondence:**Leanne Darwin – North West Health Education England, Manchester, UK

**Background**

Preoperative anaemia is a common problem [1]. It is independently associated with an increased risk of 30 day morbidity and mortality [2] and potentially treatable.

The Manchester Surgical Outcomes Project (MSOP) is a prospective observational cohort study of patients admitted to critical care following elective surgery. It is a Manchester Royal Infirmary (MRI) initiative to continuously collect peri operative morbidity data, enabling targeted quality improvement work. MSOP aimed to ascertain the prevalence of pre op anaemia, proportion of microcytosis and the rate of post operative red cell transfusion.

**Methods**

Demographic data, preoperative haemoglobin concentrations, mean corpuscular volume (MCV), and number of units of red cells transfused during hospital admission were collected on patients who underwent surgery between September 2014 and May 2015. Inclusion criteria were adult patients undergoing elective non-cardiac, non-orthopaedic surgery who were admitted to critical care following surgery. Anaemia was defined using the World Health Organisation criteria. Microcytosis was defined as an MCV < 80 fl.

**Results**

488 patients were included for analysis. 58 % (n = 282) male; 42 % (n = 206) female. 80 % (n = 391) of operations were for cancer.

Overall 39 % (n = 191) were anaemic pre operatively. 14 % (n = 27) of anaemics were microcytic.

The surgical specialties with highest prevalence of anaemia were upper GI (56 %, n = 24) and colorectal (43 %, n = 23). Hepatobiliary surgery constituted the largest surgical specialty represented (37 %, n = 180).

The overall mean average number of units of red cells transfused during the hospital admission was 1.47. The mean average red cell transfusion rate increased with severity of anaemia from 1.0 unit per non anaemic patient to 6.0 units per severely anaemic patient.

**Conclusion**

We found a prevalence of preoperative anaemia at the higher end of that found in other studies [1].

The data is limited by the absence of haematinic studies. MCV is used as a pragmatic surrogate marker for iron deficiency anaemia. It is likely that the true prevalence of iron deficiency is greater than 14 %.

Management of pre operative anaemia has been challenging at MRI due to barriers such as the limited timeframe available for pre operative optimisation prior to cancer surgery.

We have used this data to support the development and implementation of a pre operative anaemia project in pilot specialties: colorectal, hepatobiliary and upper GI surgery. We are aiming for early identification, assessment and management of anaemia including the use of intravenous iron when indicated.

**References**

1. Beattie WS, Karkouti K, Wijeysundera DN, Tait G. Risk associated with preoperative anemia in noncardiac surgery: a single center cohort study. *Anesthesiology* 2009 Mar; 110(3):574–81.

2. Musallam KM, Tamim HM, Richards T, et al. Preoperative anaemia and postoperative outcomes in on-cardiac surgery: a retrospective cohort study. *The Lancet* 2011 378(9800):1396–1407.

## A10 Preliminary results from a pilot study utilizing ears protocol in living donor nephrectomy

### **Presenting Author:**Aparna Rege^1^, Jayanth Reddy^1^, William Irish^2^, Ahmad Zaaroura^1^, Elizabeth Flores Vera^1^, Deepak Vikraman^1^, Todd Brennan^1^, Debra Sudan^1^, Kadiyala Ravindra^1^

#### ^1^Duke University Medical Center, Durham, NC, USA; ^2^CTI Clinical Trial and Consulting, Cincinnati, OH, USA

##### **Correspondence:**Aparna Rege – Duke University Medical Center, Durham, NC, USA; Jayanth Reddy – Duke University Medical Center, Durham, NC, USA

**Background/Introduction**

Gastrointestinal (GI) recovery after major abdominal surgery can be delayed from ongoing need for narcotic analgesia thereby prolonging hospitalization. Enhanced recovery after surgery (ERAS) is a multimodal perioperative care pathway designed to facilitate early recovery after major surgery by maintaining preoperative body composition and physiological organ function and modifying the stress response induced by surgical exposure.[1] Enhanced recovery programs (ERPs) in colorectal surgery have decreased the duration of postoperative ileus and hospital stay while showing equivalent morbidity, mortality and readmission rates in comparison to the traditional standard of care.[2, 3] Laparoscopic living donor nephrectomy has significantly transformed the outlook for individuals considering kidney donation.[4] However, a 30 % rate of Emergency Room visits / readmission was recorded at our center in 2014 largely from delayed GI recovery. Thus, a pilot trial to utilize ERAS protocols in living kidney donors was initiated.

**Methods**

This is a single-center retrospective analysis comparing the outcomes of the first 14 live kidney donors subjected to laparoscopic nephrectomy with ERAS protocol to 18 donors operated prior to ERAS with traditional standard of care. Both groups were matched by patient demographics. Our ERP includes reduced duration of fasting with preoperative carbohydrate loading, intraoperative fluid restriction to 3 ml/kg/hr, target urine output of 0.5 ml/kg/hr, use of sub fascial Exparel injection (Bupivacaine liposome suspension) and postoperative narcotic free pain regimen with Acetaminophen, ketorolac, tramadol.

**Results**

ERAS protocol reduced postoperative median length of stay decreased from 2.0 to 1.0 days (***P 0.001***). Overall pain scores were significantly lower in the ERAS group (peak pain score 6.50 vs 9.00 ***- p 0.001***, morning after surgery pain score 3.00 vs 7.00 - ***p 0.012***) despite absence of narcotics in the postoperative period. Average duration of surgery was shorter with the ERP as compared to the standard protocol (241 vs 277 min - ***p 0.019***). Average amount of intraoperative fluid used was significantly lower in the ERAS group in comparison to standard of care protocol (2000 ml vs 3000 ml, ***p 0.002***), without affecting the donor urine output intraoperatively or the percent change in donor serum creatinine on postop day 1 (70 vs 77, p 1.000). Incidence of delayed graft function was similar in the two groups (2 vs 1, p 0.597). A trend towards lower readmission was noted with the ERAS protocol. (2 vs 4, p 0.656). GI dysfunction was the most common reason for readmission.

**Table:** 

		**Summary Outcome Measures**		
**Variable**	**Statistics**	**Control (N = 18)**	**Treatment (N = 14)**	**P-value**
**Operative Time**	Mean(SD)	286.28(53.89)	243.00(39.88)	
	Min---Max	167.00–391.00	189.00–312.00	0.0195
	Median	277.00	241.00	
**Operative Fluid**	Mean(SD)	3049.94(841.03)	2028.57(723.01)	
	Min---Max	1500.00–4700.00	1000.00–3500.00	0.0018
	Median	3000.00	2000.00	
**Length of stay**	Mean(SD)	2.41(1.58)	1.07(0.27)	
	Min---Max	1.00–7.00	1.00–2.00	0.0012
	Median	2.00	1.00	
**Readmission**	No	12(75.0 %)	12(85.71 %)	0.6567
	Yes	4(25.0 %)	2(14.29 %)	
**Peak pain score**	Mean(SD)	8.22(1.73)	5.93(1.90)	
	Min---Max	4.00–10.00	3.00–9.00	0.0015
	Median	9.00	6.50	
**Morning pain score**	Mean(SD)	5.61(2.99)	3.00(2.25)	
	Min---Max	0.00–10.00	0.00–8.00	0.0126
	Median	7.00	3.00	
**Low pain score**	Mean(SD)	1.39(1.65)	0.50(0.94)	
	Min---Max	0.00–5.00	0.00–3.00	0.0968
	Median	1.00	0.00	
**Delayed graft function**	No	14(93.3 %)	12(85.71 %)	
	Yes	1(6.7 %)	2(14.29 %)	0.5977

**Conclusion**

Application of ERAS protocol in laparoscopic living donor nephrectomy was associated with reduced length of hospitalization. Improved pain scores resulted from intraoperative use of sub fascial Exparel and shorter duration of ileus. This is likely related to optimizing intraoperative fluids thus preventing excessive third spacing & bowel edema which prolongs gut recovery. The restricted use of intravenous fluids during donor surgery did not adversely impact recipient graft function. This study suggests that ERAS has the potential to enhance the advantages of laparoscopic surgery for live kidney donation through optimizing donor outcomes and perioperative patient satisfaction. ERP’s can further incentivize donors for undergoing laparoscopic live kidney donation.

**References**

1. Wind J, Polle SW, Fung Kon Jin PH, et al; Laparoscopy and/or Fast Track Multimodal Management Versus Standard Care (LAFA) Study Group; Enhanced Recovery after Surgery (ERAS) Group. Systematic review of enhanced recovery programmes in colonic surgery. Br J Surg. 2006; 93:800–809.

2. Lassen K, Soop M, Nygren J, et al. Consensus review of optimal perioperative care in colorectal surgery: enhanced recovery after surgery (ERAS) Group recommendations. Arch Surg 2009; 144:961–9.

3. Greco M, Capretti G, Beretta L, Gemma M, Pecorelli N, Braga M. Enhanced recovery program in colorectal surgery: a meta-analysis of randomized controlled trials. World J Surg 2014;38:1531.

4. Ratner LE, Hiller J, Sroka M, et al. Laparoscopic live donor nephrectomy removes disincentives to live donation. Transplant Proc 1997;29:3402.

## A11 Enhanced recovery after surgery: the role of the pathway coordinator

### **Author:**Deborah Watson

#### McGill University Health Centre, Montreal, Québec, CANADA

**Introduction**

Enhanced Recovery After Surgery (ERAS) pathways challenges traditional surgical care. While ERAS programs are associated with improved outcomes and cost, implementation and sustainability are recognized challenges. Since 2008, the McGill University Health Centre (MUHC) introduced ERAS elements to guide perioperative care in 12 pathways aiming to increase patient’s participation in their care. The purpose of this presentation is to describe the role, tasks and responsibilities of the ERAS nurse coordinator to guide senior management and nurses in leadership positions who wish to begin a new program or expand an existing program. Key factors that facilitated the implementation and sustainability of this organizational change at the MUHC are presented. The author will share insights learned from her experience, raise awareness of the nurse coordinator’s key role and positively recognize her institution for supporting her role and for their outstanding collaboration. It summarizes how the leadership style, the organizational culture and the type of change facilitated the implementation.

**Methods**

Growing evidence suggests using a theoretical framework or model to bring change in an organization increases the likelihood of success. The theoretical Framework based on Innovation of Diffusion Model and the Plan Do Study Act cycle guided our implementation plan. This ensured a balance between the need to provide protocol guidance using standard order sets and nursing plans with the need for efficiency and ease-of-use by the clinician.

**Results**

Our various publications show a decrease in hospital length of stay, without increasing complications or readmission rates. As the first North American ERAS Society Center of Excellence, the MUHC assists other institutions to implement the ERAS care system. The MUHC ERAS program is listed in Accreditation Canada’s Leading Practices Database.

**Conclusion**

As more and more hospitals apply lessons learned from the enhanced recovery experience in colorectal surgery to other surgical procedures, there will be an increased need for a nurse coordinator to lead this organizational change. ERAS helps to develop a culture focused on patient recovery and actively integrate patient’s participation in care. An organization wishing to start an ERAS program should set goals, report on the results, invest time to build the care pathways and provide strong leadership.

## A12 Hospitalization costs for patients undergoing orthopedic surgery treated with intravenous acetaminophen (IV-APAP) + IV opioids or IV opioids alone for postoperative pain

### **Authors:**Manasee V. Shah^1^, Brett A. Maiese^1^, Michael T. Eaddy^1^, Orsolya Lunacsek^1^, An Pham^2^, George J. Wan^2^

#### ^1^Xcenda, Palm Harbor, FL, USA; ^2^Mallinckrodt Pharmaceuticals, Hazelwood, MO, USA

##### **Correspondence:**Manasee V. Shah – Xcenda, Palm Harbor, FL, USA

**Introduction**

This study was conducted to assess the impact of intravenous acetaminophen (IV-APAP) as part of a multimodal analgesia (MMA) approach compared to IV opioid monotherapy on hospitalization costs in patients undergoing orthopedic surgery, including total knee replacement, total hip replacement, or surgical repair of hip fracture for postoperative pain management.

**Methods**

A retrospective analysis of Truven Health‘s MarketScan Hospital Drug Database (HDD), was conducted comparing patients undergoing orthopedic surgery who received multimodal postoperative pain management with combination IV APAP and other IV analgesics (IV-APAP group) to those who received only IV opioids (IV opioid group) starting on the day of surgery. Both groups could receive oral analgesics as part of their postoperative pain management regimen. Patients who underwent elective orthopedic surgery at 1 of 600 participating hospitals between January 1, 2011 and August 31, 2014, were identified and separated into postoperative pain treatment groups. Patients with evidence of substance abuse disorder and those who used methadone or buprenorphine in addition to other opioids were excluded. The 2 treatment groups were compared regarding baseline characteristics and total hospitalization costs. Differences in categorical variables were assessed using chi-square tests, while differences in continuous variables were assessed using t-tests. A multivariate sensitivity analysis was also conducted using inverse probability of treatment weighting (IPTW) with propensity scores.

**Results**

The IV-APAP (n = 33,954) and IV opioids (n = 110,300) groups were significantly different (but not clinically meaningful) across all baseline characteristics including mean age (62.1 years [IV-APAP] vs. 61.4 years [IV opioids]), percent female (56.4 % vs. 55.1 %) and if the hospital was a teaching hospital (16.6 % vs. 16.4 %); all *P* < 0.0001. Mean total hospitalization costs, which included medical costs and pharmacy costs, were statistically significantly lower for patients in the IV-APAP group as compared to patients in the IV opioids group ($12,540 vs. $13,242; *P* < 0.0001; see Table 1). Medical costs (medical/surgical supplies, laboratory testing, imaging, and other costs), drove the difference between treatment groups, encompassing $701 of the $702 between-group difference. Pharmacy costs were similar for the IV-APAP group as compared to the IV opioids group. The total cost difference remained statistically significant in the multivariate analysis, with IV-APAP utilization associated with $830 lower hospitalization costs compared to IV opioids (*P* < 0.0001).

**Conclusion**

Patients undergoing orthopedic surgery who received IV-APAP as part of MMA for postoperative pain had lower total costs than patients in the IV opioid group. This difference was mainly driven by medical costs. There was no difference observed in pharmacy costs between treatment groups.

**Table 1 (abstract A12).** Total costs for patients undergoing orthopedic surgery

	IV-APAP (n = 33,954)		IV Opioids (n = 110,300)		P Value
	Mean	SD	Mean	SD	
Total costs	$12,540	$9,564	$13,242	$35,825	<0.0001
Medical costs^a^	$12,053	$9,377	$12,754	$34,870	<0.0001
Medical/surgical supplies	$2,795	$1,870	$2,889	$5,717	<0.0001
Lab	$197	$301	$219	$1,019	<0.0001
Imaging	$91	$129	$105	$238	<0.0001
Other^b^	$8,970	$7,922	$9,541	$30,735	<0.0001
Pharmacy	$486	$488	$488	$1,120	0.6786

^a^Medical costs = medical/surgical supplies costs + lab costs + imaging costs + other costs

^b^Examples of costs included in “Other“are room and board, EKGs, oxygen, and ventilation

## A13 Development of an app for quality improvement in enhanced recovery

### **Authors:**Kirstie McPherson, Thomas Keen, Monty Mythen

#### University College London Hospital, London, UK

##### **Correspondence:**Kirstie McPherson – University College London Hospital, London, UK

**Background/Introduction**

There is compelling evidence that Enhanced Recovery Pathways (ERP’s) reduce length of hospital stay, complications and mortality. Their economic benefit is similarly impressive, saving hospital bed days and costs. Implementing and maintaining good levels of compliance with the multi-facetted pathway is challenging, and deviation from, and lack of adherence to a pathway translates to a dilution of benefits. Our organisation wanted to develop a tool to improve compliance with ERP’s, examine outcomes with greater granularity and place the patient at the very centre of their care.

**Methods**

We worked with a computer scientist to produce a bespoke “app” for our enhanced recovery colorectal patients. Checklists were embedded within the app architecture corresponding to nineteen recognised elements of an enhanced recovery pathway. In addition, the app included “goal-based” targets for patients to aid their recovery, standardised and validated outcome metrics, patient diaries and satisfaction and experience interfaces. The work represented service evaluation and did not require ethics approval. An enhanced recovery specialist nurse collected data for all ER colorectal surgical patients from May - July 2015.

**Results**

**Fig. 3 (abstract A13).**
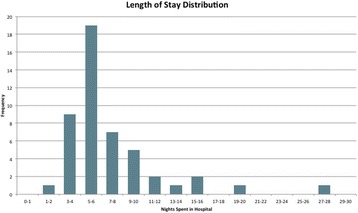
Bar chart to show distribution of length of stay for all enhanced recovery colorectal surgery patients (May-July 2015)

Data was collected for 48 patients. All patients used the patient-facing side of the app. Mean length of stay for all enhanced recovery colorectal surgery was 7.4 days *(see* Fig. 3*below)*. Analysis of data from the same time period in the previous year for colectomies and excision of rectum procedures demonstrated a reduced mean length of stay of 4.4 days in the intervention period (11.8 days versus 7.4 days). Overall compliance with the enhanced recovery pathway was 93 %. 92 % drinking, eating and mobilising on day zero, and 98 % on day 1 postoperatively.

Mean scores for satisfaction with anaesthesia and surgery were 4.2 and 4.2 respectively, out of a maximum score of 5. 93 % of patients would recommend the institution to friends and family, based on the treatment they received.

**Conclusion**

Based on our experience to date with an app that supports compliance with ERP’s, engages patients, and tracks and benchmark outcomes, we suggest this technology provides a high-value, low-cost tool to drive quality improvement. Further work is needed to evaluate the role of App’s in improving outcomes and patient experience in healthcare.

## A14 A clinical rotation in enhanced recovery pathways and evidence based perioperative medicine for medical students

### **Authors:**Alexander B Stone, Christopher L. Wu, Elizabeth C. Wick

#### The Johns Hopkins Medical Institutions, Baltimore, MD, USA

##### **Correspondence:**Alexander B Stone – The Johns Hopkins Medical Institutions, Baltimore, MD, USA

**Background**

Enhanced Recovery After Surgery (ERAS) pathways are evidence based, multidisciplinary perioperative bundles of interventions that have been shown to reduce length of stay, complications and costs and improve the patient experience following surgery. They are widely adopted in Europe and Canada and are increasingly being implemented in the United States. Currently, there are no published programs for educating medical students about ERAS programs. In the basic surgery clerkship at our institution, there were limited opportunities for learning about ERAS programs because of an emphasis on spending time in the operating room. Additionally, few students had the opportunity to rotate on service lines that featured an ERAS program. A survey of medical students done in the United Kingdom, where ERAS is widely implemented, found that only 14 % of students had heard of ERAS [1]. We sought create a clinical rotation that gave students the opportunity to engage with the many facets of our ERAS program at the three sites within the Johns Hopkins Medicine system.

**Methods**

We developed a four-week curriculum that had three core objectives. First, the student was to engage with the wide variety of allied health providers that participate in the ERAS program. The goal was to gain a 360 degree appreciation for the patient’s surgical journey; from the initial office visit, to follow up home care nursing. Second, the student was to become familiar with the evidence base behind the ERAS program. The student was expected to prepare weekly presentation on one aspect of the ERAS pathway. Last, the student was expected to engage in the clinical research associated with the ERAS pathway. The pilot rotation was set for February 2016.

**Results**

Over the course of the month long curriculum, the student was able to able to interact with a wide range of allied health providers, including; surgeons, anesthesiologists, physician assistants, CRNAs, nursing staff on the surgical floors, as well as home visits with the home care nursing team. This allowed for a more complete view of the surgical journey and emphasized the wide range of providers that need to collaborate for excellent perioperative care to be successful. The student was able to rotate a three different sites, Johns Hopkins Hospital, Bayview Medical Center, and Sibley Memorial Hospital, and appreciate how the ERAS programs were implemented in unique ways at each site. Having a student rotate through each of the site improved collaboration between the ERAS programs at different sites. The weekly PowerPoint presentations were archived and kept for future students to reference as they go through the rotation.

**Conclusion**

This initial trial of an ERAS elective for Medical Students provided a global view of the surgical journey and imbued a greater appreciation for how allied medical professionals come together to provide excellent evidence based perioperative care. We look forward to offering this elective to future students as well as preparing a didactic session to be integrated into the core surgery clerkship.

**Reference**

1. McLennan E, Renwick A, Moug SJ. The current undergraduate medical school curriculum needs to improve awareness of enhanced recovery after surgery. .Color. Dis. Off. J. Assoc. Coloproctology Gt. Br. Irel.; 2014;16:927–929.

## A15 Enhanced recovery after surgery (ERAS) implementation in abdominal based free flap breast reconstruction

### **Authors**: Rachel A. Anolik, Adam Glener, Thomas J. Hopkins, Scott T. Hollenbeck, Julie K. Marosky Thacker

#### Duke University School of Medicine, Durham, NC, USA

##### **Correspondence:**Rachel A. Anolik – Duke University School of Medicine, Durham, NC, USA

**Background**

Breast reconstruction continues to be important for many women with breast cancer. Following a mastectomy, the breast may be reconstructed with either implants or the patient’s own tissue. The benefit of breast reconstruction with tissue is the more natural feel and long term durability in comparison to implants. The downside of a tissue reconstruction is the donor site scar and perceived complexity and pain associated with this approach. Through refinements in technique, breast reconstruction with abdominal tissue has become more streamlined and less invasive. As the techniques have evolved so has the management of the patient in the perioperative period. Enhanced Recovery After Surgery (ERAS) initiatives have been implemented in many hospitals with the aim to improve post-operative physiologic function and recovery. In this study, we sought to compare the outcomes of patients undergoing abdominal based free flap breast reconstruction before and after implementation of the ERAS protocol.

**Methods**

This is an IRB approved retrospective study which involves analysis of data extracted from chart review. Evaluable subjects were defined as those who have undergone abdominal free flap breast reconstruction at Duke University Hospital, identified using the CPT code 19364. Patients with pre-existing psychiatric and chronic pain conditions were excluded. The ERAS protocol was implemented in May of 2015. For this study, data was collected from 10/1/2014 through 1/1/2016 in order to include patients before and after implementation of ERAS protocol. Patient demographics, perioperative surgical and anesthesia data, need for analgesics, and complications were collected and summarized. Statistics were done with JMP and Microsoft excel 2013.

**Results**

There were 17 patients in the control and 21 patients in the ERAS group. Both groups had similar age, race and BMI. The patients in the ERAS group had a significantly reduced length of stay (LOS) (3.8 v. 4.76 days, p = 0.0003, *t*-test). There was no significant difference in 24 hr morphine equivalent dosage (MED) (34.17 vs. 22.2 mg, p = 0.26, *t*-test). However, intravenous (IV) pain medication usage was reduced in ERAS patients (14/21 v. 17/17 patients, p = 0.0049, *t*-test) and patients had earlier return of bowel function (POD 2 v. 3.5, p = 0.002, *t*-test). There was no significant difference in the rate of flap loss between groups (6.45 % ERAS v. 3.57 % control, p = 0.617, *t*-test).

**Conclusion**

Implementation of an ERAS protocol for abdominal free flap breast reconstruction at a tertiary medical center was associated with a significantly reduced LOS, decreased IV narcotic pain medication usage and earlier return of bowel function. This is consistent with results seen in colorectal surgery and suggests that this program could be instituted nationwide for standardization of breast reconstruction with improved outcomes.
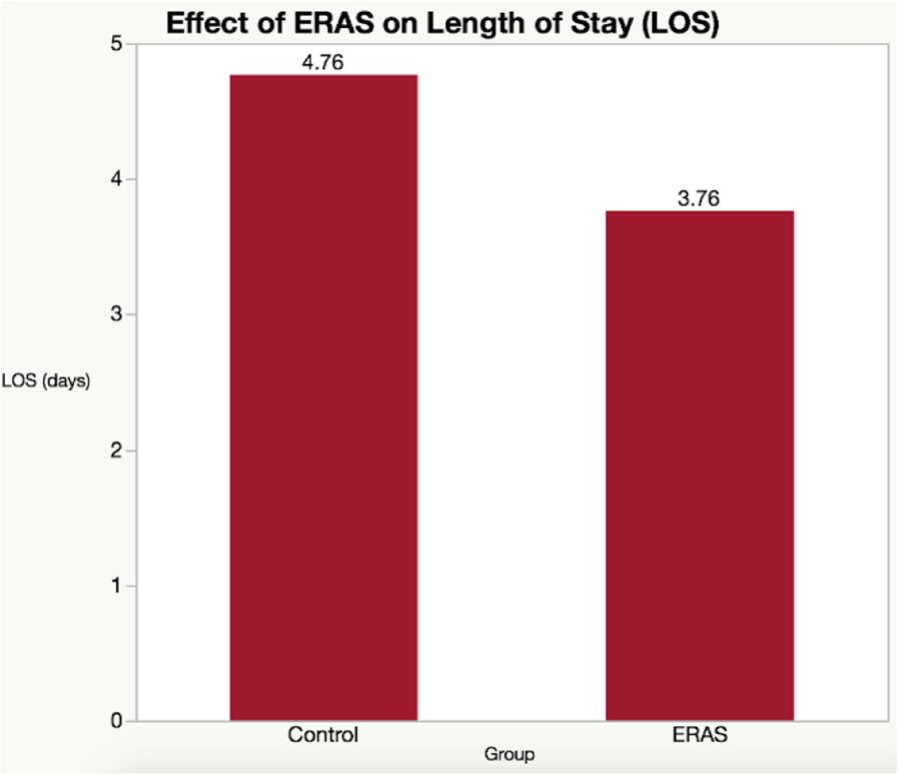


## A16 How the implementation of an enhanced recovery after surgery (ERAS) protocol can improve outcomes for patients undergoing cystectomy

### **Authors:**Tracey Hong^1^, Andrea Bisaillon^1^, Peter Black^2^, Alan So^2^, Associate Professor, Kelly Mayson^1,2^

#### ^1^Vancouver General Hospital, Vancouver, British Columbia, Canada; ^2^University of British Columbia, Vancouver, British Columbia, Canada

##### **Correspondence:**Tracey Hong – Vancouver General Hospital, Vancouver, British Columbia, Canada

**Background**

Despite improvements in surgical techniques and perioperative care protocols, radical cystectomy (RC) is still associated with higher morbidity than other urological procedures. In our hospital, RC had accounted for 38.2 % of postoperative complications but only 13.6 % of the total urological case volume as demonstrated in the risk-adjusted reports (07/2011-06/2014) from the American College of Surgeons National Surgical Quality Improvement Program (ACS NSQIP). Morbidity impacts patient’s safety and experience, increases hospital length of stay and health care costs.

The multimodal evidence-based perioperative care pathway Enhanced Recovery After Surgery (ERAS) offers opportunity to reduce complications after major surgery, which has been validated in the elective colorectal cases in our hospital.

**Methods**

A multidisciplinary team was formed in April 2014. A project charter and an implementation plan were initiated. ERAS documents such as order sets, patient education booklet and clinical pathway were developed. Comprehensive and ongoing education on ERAS principles and our local experience were shared with the surgical staff. In October 2014, we implemented our ERAS protocol to all Urology patients undergoing elective radical cystectomy surgery. Real time auditing of compliance with the 21 ERAS components and measuring of post-operative complications, hospital length of stay and readmission as defined by the American College of Surgeons National Surgical Quality Improvement Program (ACS NSQIP) were started immediately post ERAS implementation. Results for pre- and post-ERAS cohorts were compared, using Fisher’s exact test. The goal was to decrease the overall morbidity for target patient population by 50 % by September 2015.

**Results**

For the first 13 months post implementation, 91 consecutive radical cystectomy patients had been enrolled in the ERAS program. Patient demographics and co-morbidity counts were similar in both cohorts. Process measures showed that the pre-operative and intra-operative components had met and sustained our goal of a minimum of 80 % compliance within first month post implementation. Post-operative components have been the slowest to change, but they are trending towards our goal. The rates of post-operative overall morbidity fell from 31.3 % to 18.7 % (p = 0.059). UTI declined from 10 % to 1.1 % (p < 0.05), which was statistically significantly lower post implementation as seen in Table 2.

**Conclusion**

Teamwork and communications of a multidisciplinary team are crucial to a culture of patient safety. Use of real-time auditing and the Plan-Do-Study-Act (PDSA) cycles enhance our rate of improvement. Aggregation of marginal gains can result in dramatic improvements in patient outcomes, which has proven in elective radical cystectomy cases after ERAS implementation in our hospital.

**Table 2 (abstract A16).** Patient Outcomes Pre- and Post-ERAS Implementation

	Pre-ERAS	Post-ERAS	P Values
	May 2011 – Sept. 2014	Oct. 2014 – Nov. 2015	
	n = 90	n = 91	
Age (mean)	69	67	
NSQIP Co-morbidity Count (mean)	1.1	1.06	
NSQIP 30-day Morbidity Incidence	31.1 %	↓18.7 % (40 % reduction)	p = 0.059
Urinary Tract Infections (UTI)	10 %	↓1.1 %	p < 0.05
Transfusion (72 hr of OR start time)	43.3 %	↓29.7 %	p = 0.0695
Readmissions Within 30 Days	16.7 %	↓12.1 %	
Median LOS Post-OR Days	7.5	↓7	

## A17 Use of an app to improve patient engagement with enhanced recovery pathways

### **Authors:**Kirstie McPherson, Thomas Keen, Monty Mythen

#### University College London Hospital, London, UK

##### **Correspondence:**Kirstie McPherson – University College London Hospital, London, UK

**Background**

Patient engagement with enhanced recovery pathways (ERP’s) forms up to thirty percent of a pathway’s portfolio of compliance elements. This includes consumption of preoperative carbohydrate drinks, early mobilisation and resumption of oral intake. In addition to these explicit elements; by making transparent the goals of a pathway, the patient may implicitly improve their pathway compliance, by challenging and engaging with clinicians on their progress and attainment of recovery milestones.

**Methods**

**Fig. 4 (abstract A17).**
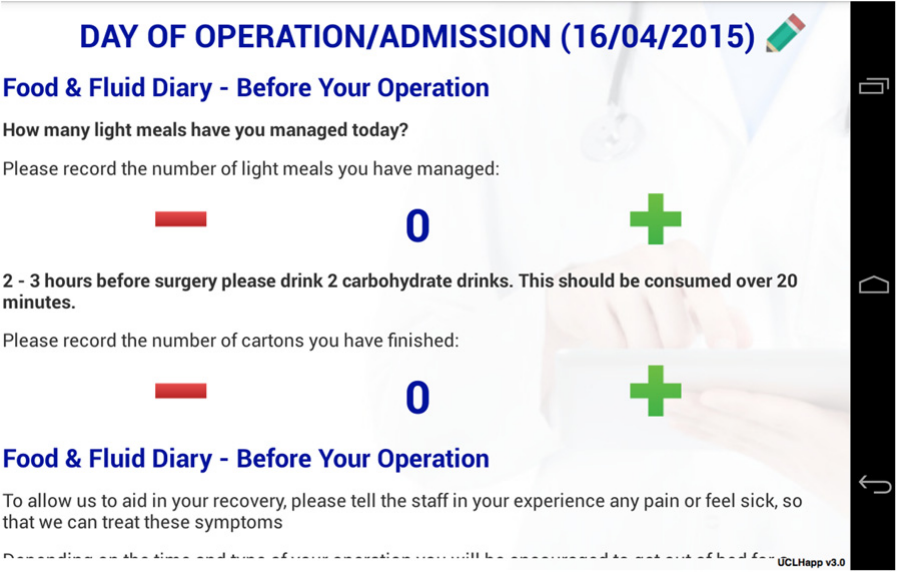
Typical screenshot of patient-facing interaction with colorectal ERP

We used an app to track compliance with elements of enhanced recovery for colorectal surgery. The app contained both patient-facing and clinician-facing domains. From May - July 2015, all patients enrolled on a colorectal ERP in our institution (n = 48) used the app. As they moved through the pathway, the app provided prompts and opportunities to remind and engage with them on the anticipated goals of recovery, explicitly making clear expectations such as postoperative exercise. (See Fig. 4)

**Results**

100 % (n = 48) used the app. Compliance with patient-centred elements of the pathway was 93 % (i.e. for preoperative CHO drinks, mobilisation and resumption of oral intake). This is compared to pathway compliance of 19 % for preoperative CHO drinks, prior to the introduction of the app.

**Conclusion**

Compliance with the pathway since the introduction of the app has improved to 93 %. By putting the patient at the centre of their care and making transparent the goals of recovery, compliance and outcomes are improved. We firmly believe that patients represent a powerful driver for improved delivery of healthcare. More efforts should be made to make patient information and goals of recovery readily available to patients. The architecture of apps provides a useful platform on which to pursue this venture.

## A18 Effect of an enhanced recovery after surgery pathway for living donor nephrectomy patients

### **Authors:**Adam B. King, Rachel Forbes, Brad Koss, Tracy McGrane, Warren S. Sandberg, Jonathan Wanderer, Matthew McEvoy

#### Vanderbilt University Medical Center, Nashville, TN, USA

##### **Correspondence:**Adam B. King – Vanderbilt University Medical Center, Nashville, TN, USA

**Introduction**

Perioperative care in the United States is often costly and fragmented. A number of studies have demonstrated that enhanced recovery after surgery (ERAS) programs reduce morbidity, hospital costs, and length of hospital stay [1], however there have been no documented ERAS protocols for living donor nephrectomy patients assessing effectiveness in this patient population. The concept of the Perioperative Surgical Home (PSH) advances upon ERAS by placing these multi-component care pathways into a system of care that spans the period from decision to discharge. In partnership with kidney transplant surgeons at our institution, we developed and incorporated an ERAS pathway for living donor nephrectomies through our existing PSH known as our Perioperative Consult Service (PCS).

**Methods**

After IRB approval, records were obtained for all living donor nephrectomies (2/07/2013 - 1/28/2016). All patients undergoing a living donor nephrectomy performed by a kidney transplant surgeon as identified by their primary procedural surgical code were included. Post implementation of our ERAS pathway, all living donor nephrectomy patients were included unless had allergy to medication in protocol, contraindication to regional anesthesia, or patient refusal. Patient ASA classification, gender and BMI were obtained, along with morphine equivalents. Length of stay was abstracted from hospital billing records.

**Results**

142 charts were reviewed; 113 were pre implementation of our protocol (2/07/2013 – 7/27/2015) and 29 were post implementation (7/28/2015 – 1/28/20016). There was no difference in ASA classification or gender, BMI, or preoperative morphine equivalents between the two groups. All procedures were performed laparoscopically. Intraoperative and Post Anesthesia Care Unit morphine equivalents were significantly reduced between pre and post implementation of protocol (39.21 vs 4.38, P <0.001 and 7.24 vs 2.54, p < 0.001 respectively). Mean and median length of stay was decreased between pre and post implementation phases: 2.84 vs 2.27, p < 0.001 and 2.48 vs 2.34, respectively. Prior to implementation, only 55 % of patients were discharged prior to POD3, whereas after implementation, 93 % were discharged prior to POD (P < 0.001), with some patients going home on POD1. Readmission events within 30 days, although higher in the post implementation group, were extremely low for both groups: 0/113 pre implementation vs 2/29 post implementation (one for nausea and one for abdominal pain) and emergency department visit events were 1/113 pre implementation (fever) vs 2/29 post implementation (one for nausea and one for chest pain). There were no rapid responses or postoperative ICU admissions in either group.

**Conclusion**

The living donor nephrectomy ERAS pathway development and implementation by our PCS significantly shortened median length of stay and decreased perioperative opiate use in living donor nephrectomy surgery patients. Of note, if we are able to sustain these changes, we will be able to liberate approximately 40 bed-days per year even from this lower volume service. Future directions will involve applying ERAS principles to kidney transplant recipients.

## A19 Introduction and implementation of an enhanced recovery program to a general surgery practice in a community hospital

### **Authors:**Patrick Shanahan^1^, John Rohan^1^, Desirée Chappell^1^, Carrie Chesher^2^

#### ^1^Anesthesiologists Consultants Enterprises, PLLC, Louisville, KY, USA; ^2^Norton Audubon Hospital, Louisville, KY, USA

##### **Correspondence:**Patrick Shanahan – Anesthesiologists Consultants Enterprises, PLLC, Louisville, KY, USA

**Background**

The practicing general surgeons at this hospital had length of stay greater than NSQIP averages for DRGs 329–331. This varieance was the primary motivation for an enhanced recovery (ER) initiative. Eight general surgeons, practicing in three separate practice groups, were introduced to the ER concepts with the desire for the entire general surgery team to adopt and implement the designed pathway. Initially, only one surgeon became an early adopter. However, after preliminary results were shared with the other surgeons, two additional surgeons became early adopters as well. The results of the early adopting surgeons demonstrated consistency with previously published positive results from ER. All surgeons fully adopted the enhanced recovery pathway into their practice within three months maintaining consistent results.

**Methods**

The anesthesia team modeled the ER pathway after accepted and published ER guidelines [1]. The pathway was designed over three months and initiated in January 2015. Individual surgeons were presented with the evidence, their LOS data for colorectal patients and predetermined order sets to manage the ER patients. Participation was determined by their compliance with the pathway for the non-emergent colorectal surgical patients. The authors provided ER education for surgeons, anesthesia, nursing (all phases of care), managers, administration and other adjunct facility departments. The authors closely followed the ER patients during the earlier phases of implementation to ensure compliance. Slight modifications to the protocol, using feedback from all practitioners, occurred in the early stages of implementation. The continual process of audit and refinement ensure compliance and any needed improvements within the pathway.

**Results**

**Fig. 5 (abstract A19).**
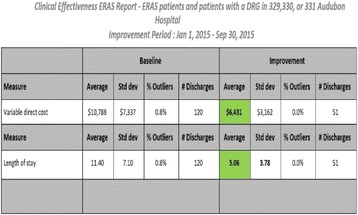
ᅟ

**Fig. 6 (abstract A19).**
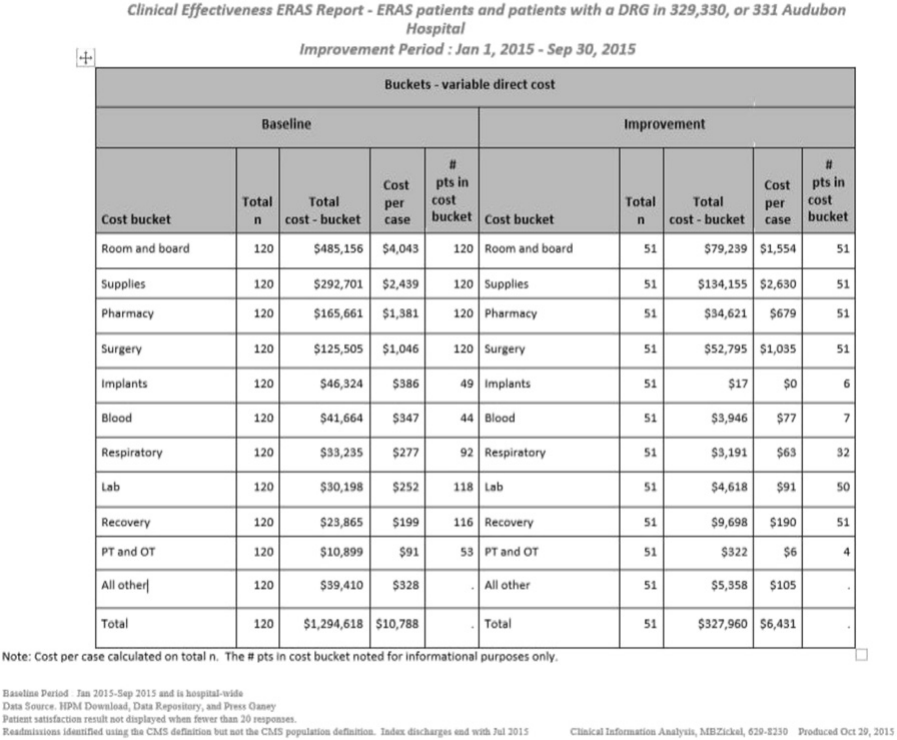
ᅟ

Adoption of an ER program by the institution and all of the general surgeons was realized and accepted within the first several months of implementation. Within the first nine months, the ER program had a greater than 50 % reduction in LOS and a variable direct cost reduction of $4357 per case (Fig. 5). In addition, the reduction in other cost buckets resulted in substantial savings for the hospital (Fig. 6).

**Conclusion**

The general surgeons adopted the ER program into their practice during 2015 and are now active, enthusiastic participants within this initiative. Initially, several of the surgeons disagreed with the well-established ER principles, but after use of the pathway by competing practitioners and the revelation of the results, adoption was universal. The use of evidence based practice information presented by a team of dedicated professionals can achieve rapid positive results in cost savings, reduced length of stay and broad based surgical adoption.

**References**

1. Varadhan KK1, L. D. (2010 Jul;26(3):). Enhanced recovery after surgery: the future of improving surgical care. *Crit Care Clin.* 527–47.

## A20 “Get fit” for surgery: benefits of a prehabilitation clinic for an enhanced recovery program for colorectal surgical patients

### **Authors:**Susan VanderBeek, Rebekah Kelly

#### Beaumont Health System, Troy, MI, USA

##### **Correspondence:**Susan VanderBeek

**Purpose:** The purpose of this project is to implement an Enhanced Recovery After Surgery (ERAS) clinic at Beaumont Health System-Troy campus for patients undergoing elective colorectal surgery.

**Introduction**

Enhanced Recovery After Surgery (ERAS) is an innovative method of patient care management designed to accelerate patient recovery following surgery and decrease post-operative complications. ERAS uses a multi-disciplinary approach to surgical care designed to improve patient readiness, enhance patient recovery and increase patient satisfaction throughout the entire surgical experience by allowing the patient to actively participate in their own preparation and recovery periods. By utilizing a collection of ERAS evidence-based strategies, we can successfully contribute to our patient’s recovery without compromising their safety. Currently there is no standardized means of managing expectations of patients undergoing complex surgical procedures. Efforts in the past have failed due to overly complex and often contradictory educational efforts, and have led to patient confusion, dissatisfaction, and poor compliance. Using established standards for healthcare literacy as well as evidence based perioperative preparatory instructions, we seek to properly prepare our patients for colorectal surgery. We anticipate that this standardized and evidence based approach will improve satisfaction, reduce outcome variability, and shorten length of stay.

**Method**

To implement this program, a multidisciplinary team of experts was assembled to drive this project forward. Team members meet monthly and include: surgeons, anesthesiologists, nurse educator, quality nursing, floor nursing, pharmacy, librarian, CRNAs, administrative managers, and a project manager. Our comprehensive ERAS care pathway includes many of the established ERAS protocols including: nutrition optimization, strength and conditioning, appropriate use of pre-medication, bowel preparation and optimization, goal directed fluid therapy, anesthetic optimization, multimodal analgesia, glycemic control, nausea and vomiting control, early feeding and bowel stimulation, early mobilization and conditioning, multimodal analgesia, glycemic control, and education and expectation management

After 1 year of the Enhanced Recovery Program we decided to open multi-disciplinary pre-operative clinic for surgical patients. The clinic is called the STTAR clinic which is an acronym for Surgical Testing and Teaching for an Accelerated Recovery. It serves as a “1 stop shop” for patients and all pre-operative activities are able to get accomplished during this single clinic visit. This serves as a tremendous satisfier for the patient and helps to eliminate the last minute scramble that often happens in the pre-operative unit on the day of surgery. A pre-operative patient clinic visit is multi-disciplinary in nature and includes: history and physical from a PA, consult from an anesthesiologist, drawing of necessary labs by RN, EKG and other diagnostics, dietary consult as needed, a goody bag with nutritional drinks, incentive spirometer, pedometer and patient educational booklet and visit from the ostomy nurse as needed.

**Results**

Based on data examining our first 150 ERAS cases in the clinic since July 2015, we have seen a reduction in length of stay from 5.05 days to 4.30 days with a decrease in direct cost from $8,171 to $7,245. We have also seen a reduction in surgical site infections and a reduction in readmissions. We have received very positive feedback from patients based on patient surveys. We anticipate seeing similar results as the project expands.

**Future Plans**

Our STTAR clinic continues to grow with not only colorectal surgery but we have expanded to Urology and have included cystectomies. Our future plan is to expand to other disciplines including orthopedics and spine surgery.

## A21 Evaluation of gastrointestinal complications following radical cystectomy using enhanced recovery protocol

### **Authors:**Siamak Daneshmand^1^, Soroush T. Bazargani^2^, Hamed Ahmadi^3^, Gus Miranda^4^, Jie Cai^4^, Anne K. Schuckman^4^, Hooman Djaladat^4^

#### ^1^Keck Hospital of USC, Los Angeles, CA, USA; ^2^NYU Langone Medical Center, New York City, NY, USA; ^3^Oregon Health and Science University, Portland, OR, USA; ^4^University of Southern California, Los Angeles, CA, USA

##### **Correspondence:**Siamak Daneshmand – Keck Hospital of USC, Los Angeles, CA, USA

**Introduction and objectives**

Gastrointestinal (GI) complications are common after radical cystectomy (RC) and urinary diversion (UD). Enhanced recovery after surgery (ERAS) protocols aim to optimize GI function, and predicated on avoiding bowel preparation and nasogastric tubes, early feeding, focus on nonnarcotic pain management and the use of cholinergic and mu-opioid antagonists. We evaluated whether our institutional ERAS protocol was associated with changes in GI function and complication rates in the first 30 days after RC and compared them to our previous traditional method of postoperative care.

**Methods**

Using our bladder cancer IRB approved database, we identified 377 consecutive patients who underwent open RC and UD using our ERAS protocol from 5/2012 to 12/2015. Also, we identified a control group who were treated with traditional (non-ERAS) post-operative care using our institutional bladder cancer database (2003 to 2012). We compared bowel activity in the postoperative period as well as GI complications for the first 30 days. Postoperative ileus (POI) was defined as oral intake intolerance that persisted beyond 5 days after surgery or by nausea and emesis with accompanied abdominal distention requiring GI rest, or a nasogatric tube at any time postoperatively. Complications were recorded based on Clavian-Dindo system.

**Results**

A total of 145 patients on ERAS arm and 144 matched controls were included in the study. Median time from surgery to first bowel movement was 2 days in the ERAS arm and 5 days in the control group (p = 0.003). GI complications within 30 days occurred in 19 (13 %) patients with the ERAS protocol and 40 (27 %) of controls (p < 0.001); the most common GI complication was postoperative ileus (POI)/partial small bowel obstruction (pSBO) in both groups (7 % vs. 23 %; p < 0.001) (Table 3). Nasogastric or gastric tube placement was required in 11 patients (7 %) in the ERAS arm compared with 25 patients (17 %) controls (p = 0.01), while Total parenteral nutrition was required in one (0.6 %) patient in the ERAS cohort and 8 (6 %) controls (p = 0.02). Median length of hospital stay (LOS) was significantly shorter in ERAS cohort compared to controls [4 (range, 3–16) d vs. 9 (range, 5 – 23) d; p < 0.001].

**Conclusions**

Our institutional ERAS protocol for RC was associated with significantly shorter time to bowel function recovery, fewer GI complications, and a shorter LOS. This protocol should be considered to reduce GI morbidity associated with open RC.

Keywords: bladder cancer, cystectomy, enhanced recovery, GI complications

**Table 3 (abstract A21).** GI-related complications in patients on ERAS protocol vs. matched non-ERAS

	ERAS patients (n = 145)	Non-ERAS controls (n = 144)	P value
30-day GI complication rate (%)	19 (13)	40 (27)	0.003
Ileus/pSBO (%)	10 (7)	34 (23)	<0.001
Intractable nausea/vomiting (%)	4 (3)	3 (2)	0.5
Need for NG/G-tube (%)	11 (7)	25 (17)	0.012
Need for TPN (%)	1 (<1)	8 (6 %)	0.02
C. Diff diarrhea (%)	3 (2)	1 (<1)	0.3
30-d readmission rate due to GI complication (%)	2 (10)	2 (5)	0.1

## A22 Impact of a novel diabetic management protocol for carbohydrate loaded patients within an orthopedic ERAS protocol

### **Authors:**Volz L, Milby J

#### Thompson J Dunes Surgical Hospital, Dakota Dunes, SD, USA

##### **Correspondence:**Volz L – Thompson J Dunes Surgical Hospital, Dakota Dunes, SD, USA

**Background**

Carbohydrate loading prior to surgical procedures has been proven to decrease peri-operative insulin resistance leading to decreased surgical complication rates. Applying this process to patients with diabetes has been questioned due to elevated preoperative blood glucose levels with standard diabetic medication management. It is postulated that maintaining diabetic patients on their standard regimen of hypoglycemic medications will lead to improved perioperative blood glucose (BG) levels. We have implemented a diabetic medication protocol (DMP) for Type I/II diabetics where patients undergo carbohydrate loading and continue their standard hypoglycemic medication regimen through the morning of surgery.

**Methods**

We initiated an Enhanced Recovery after Surgery (ERAS) protocol in October 2014 where all patients undergoing total joint replacement (TJR) received carbohydrate loading over 12 hours prior to the start of surgery. The patients were provided with 3 bottles of a maltodextran based carbohydrate drink to consume over the 12 hours prior to surgery with the last drink taken 3 hours prior to surgical start time. Diabetics were asked to hold any oral hypoglycemic medications and take half their usual insulin dose the morning of surgery. On August 1, 2015 we implemented a DMP where carbohydrate loaded diabetics would continue, without modification, their diabetic medications until arrival at the hospital the day of surgery. We performed a retrospective review of 57 consecutive diabetic TJR patients from August 1 to December 31, 2015 compared with 25 consecutive diabetic TJR patients prior to implementing the DMP.

**Results**

A total of 82 diabetic patients undergoing TJR were reviewed. 25 patients prior to implementation and 57 patients after implementation were reviewed. 12 patients prior to implementation and 11 patients after implementation were excluded due to failure to comply appropriately with medication and or carbohydrate instructions Data was extracted on preoperative, intraoperative, recovery room and postoperative BG levels.

**Table:** 

	BG BEFORE DMP			BG AFTER DMP		
	<80	80–200	>200	<80	80–200	>200
Preoperative	0	10	3	4	30	12
Recovery Room	1	10	2	5	40	1
POD#0 4 PM	0	8	5	1	29	16
POD#0 9 PM	0	9	4	0	32	14
POD#1 7 AM	1	10	1	0	37	9
POD#1 11 AM	0	4	8	0	27	17
POD#1 4 PM	0	5	6	0	35	9
POD#1 9 PM	0	5	6	0	27	18

**Conclusions**

We have been able to show that diabetic patients may safely receive carbohydrate loading prior to TJR. Diabetic patients undergoing carbohydrate loading prior to surgery are able to safely continue their diabetes medications prior to surgery without a significantly higher incidence of perioperative hypoglycemia. The DMP did not lead to a decrease in the number of patients presenting with hyperglycemia prior to surgery though BG levels were significantly improved on POD#1 at 4 PM. The overall number of complications was very low in both groups, therefore the impact of this protocol on surgical outcomes has yet to be determined. This documents the safety of carbohydrate loading diabetic patients prior to surgery as well as continuing diabetic medications until surgery. This practice should be evaluated further to determine the impact of this protocol on surgical outcomes.

## A23 Institution of a patient blood management program to decrease blood transfusions in elective knee and hip arthroplasty

### **Presenting Author:**Opeyemi Popoola, Tanisha Reid, Luciana Mullan, Mehrdad Rafizadeh, Richard Pitera

#### Saint Barnabas Medical Center, Livingston, NJ, USA

##### **Correspondence:**Opeyemi Popoola – Saint Barnabas Medical Center, Livingston, NJ, USA

**Background**

Major orthopedic surgery is associated with an anticipated level of high blood volume loss.[1,2] Pre-operative anemia is an independent prognostic factor of increased mortality and morbidity following orthopedic surgery.[1,3] It has been shown that approximately 40 % of patients evaluated prior to elective orthopedic surgeries are anemic (women Hb <12 g/dl, men Hb < 13 g/dl).[1] Pre-operative anemia is a major predictor of allogeneic blood transfusion (ABT).[2,3] ABT during the perioperative period is known to be associated with increased rate of infections, transfusion reactions, perioperative mortality and increased length of stay.[1,2,3] Our effort aimed at reducing the incidence of blood transfusions during elective joint arthroplasty.

We instituted a Patient blood management (PBM) program as a component of our Perioperative Surgical Home with the goal of improving patient outcomes and reducing the incidence of perioperative anemia in joint arthroplasty patients. The goals of our PBM program are to (1) Identify and treat pre-operative anemia, (2) Reduce autologous blood transfusions, (3) Reduce blood loss during surgery, (4) Reduce allogeneic blood transfusions, (5) Increase tolerance to anemia and adaptation of transfusion triggers.

**Methods**

Preoperative, intraoperative and postoperative PBM protocols were implemented for all patients undergoing elective knee and hip arthroplasty. Preoperatively, patients were seen approximately 30 days prior to surgery for clinical evaluation and assessment, which comprised of screening for bleeding and coagulation risk as well as anemia. Patients were treated with one or more of the following: IV iron, vitamin supplementation or erythropoietin stimulating agents. Autologous blood donation was eliminated. Intraoperatively, the use of cell savage, hemostatic agents and antifibrinolytics was instituted. Post operatively, post-surgical anemia was assessed and treated with IV iron. Blood products ordered for joint arthroplasty patients required approval from anesthesia prior to transfusion. Transfusion triggers dropped to 7 g/dl in non-cardiac patients and at 8 g/dl in cardiac patients.

**Results**

Since implementing the PBM program, blood utilization has decreased drastically. There has also been a corresponding reduction in length of stay. At the initiation of the program in 2013, the rate of transfusion in total knee arthroplasty was 16.50 %. There has since been a significant decrease to 8.22 % in 2014 and 2.87 % in 2015. In total hip arthroplasty, the average rate of transfusion was 24.44 % in 2013, with a decrease to 13.03 % in 2014 and 10.64 % in 2015. In 2013, the average length of stay was 3.20 and 3.48 for total knee and hip arthroplasty patients with a drop to 2.86 and 3.05 respectively in 2015.

**Conclusion**

Implementation of a patient blood management program is an effective way to treat preoperative anemia, reduce allogeneic blood transfusions and improve patient outcomes and risk, while reducing length of stay and reducing cost.

**References**

1. Layton LL, Rubin LE, Sweeney JD. Advanced Blood Management Strategies for Elective Joint Arthroplasty. RI Med J 2013; 96 (3): 23–25

2. Kotze A, Carter LA, Scally AJ. Effect of a patient blood management programme on preoperative anemia, transfusion rate, and outcome after primary hip or knee arthroplasty: a quality improvement cycle. Br J Anaesth. 2012; 108 (6): 943–952.

3. Frances RC, Melchor RJ, Calvo VecinoIs JM. Is it time to integrate patient blood management in ERAS guidelines? Rev Esp Anestesiol Reanim. 2015; 62 (2): 61–63.

