# Erratum to: Proceedings of the American Society for Enhanced Recovery/Evidence Based Peri-Operative Medicine 2016 Annual Congress of Enhanced Recovery and Perioperative Medicine: Washington, D.C., USA, 20–22 April 2016

**DOI:** 10.1186/s13741-016-0050-3

**Published:** 2016-10-07

**Authors:** Charles R. Horres, Mohamed A. Adam, Zhifei Sun, Julie K. Thacker, Timothy E. Miller, Stuart A. Grant

**Affiliations:** Duke University School of Medicine, Durham, NC USA

## Erratum

Following publication of the original article (Horres et al. 2016) it was brought to our attention that one of the author names for A1 (*Effects of enhanced recovery pathways on renal function*) was inaccurately represented due to a typo. Please therefore note that the author name Timothy J. Miller should instead read as Timothy E. Miller. We apologise for any inconvenience caused.
